# Supplementary material for: Reduced body sizes in climate-impacted Borneo moth assemblages are primarily explained by range shifts
Source: Nat Commun. 2019 Oct 10;10:4612. doi: 10.1038/s41467-019-12655-y (PMC6787050; doi:10.1038/s41467-019-12655-y)
Supplement: Supplementary file 1 — Reporting Summary [file 41467_2019_12655_MOESM1_ESM.pdf]

nature research

Corresponding author(s): I-Ching Chen
Chuan-Kai Ho

Last updated by author(s): Sep 3, 2019

Reporting Summary

Nature Research wishes to improve the reproducibility of the work that we publish. This form provides structure for consistency and transparency in reporting. For further information on Nature Research policies, see Authors & Referees and the Editorial Policy Checklist.

Statistics

For all statistical analyses, confirm that the following items are present in the figure legend, table legend, main text, or Methods section.

n/a	Confirmed	
☐	☒	The exact sample size (n) for each experimental group/condition, given as a discrete number and unit of measurement
☐	☒	A statement on whether measurements were taken from distinct samples or whether the same sample was measured repeatedly
☐	☒	The statistical test(s) used AND whether they are one- or two-sided<br>*Only common tests should be described solely by name; describe more complex techniques in the Methods section.*
☐	☒	A description of all covariates tested
☐	☒	A description of any assumptions or corrections, such as tests of normality and adjustment for multiple comparisons
☐	☒	A full description of the statistical parameters including central tendency (e.g. means) or other basic estimates (e.g. regression coefficient) AND variation (e.g. standard deviation) or associated estimates of uncertainty (e.g. confidence intervals)
☐	☒	For null hypothesis testing, the test statistic (e.g. F, t, r) with confidence intervals, effect sizes, degrees of freedom and P value noted<br>*Give P values as exact values whenever suitable.*
☒	☐	For Bayesian analysis, information on the choice of priors and Markov chain Monte Carlo settings
☐	☒	For hierarchical and complex designs, identification of the appropriate level for tests and full reporting of outcomes
☐	☒	Estimates of effect sizes (e.g. Cohen's d, Pearson's r), indicating how they were calculated

Our web collection on statistics for biologists contains articles on many of the points above.

Software and code

Policy information about availability of computer code

Data collection	n/a
Data analysis	Statistical analysis and sub-sampling were conducted using R version 3.5.0

For manuscripts utilizing custom algorithms or software that are central to the research but not yet described in published literature, software must be made available to editors/reviewers. We strongly encourage code deposition in a community repository (e.g. GitHub). See the Nature Research guidelines for submitting code & software for further information.

Data

Policy information about availability of data

All manuscripts must include a data availability statement. This statement should provide the following information, where applicable:

- Accession codes, unique identifiers, or web links for publicly available datasets
- A list of figures that have associated raw data
- A description of any restrictions on data availability

The source data to generate all figures (except conceptual Figure 2) and tables in the main text and the supplementary information are available in Figshare with data DOI: 10.6084/m9.figshare.9728411.

Field-specific reporting

Please select the one below that is the best fit for your research. If you are not sure, read the appropriate sections before making your selection.

☐ Life sciences ☐ Behavioural & social sciences ☒ Ecological, evolutionary & environmental sciences

Ecological, evolutionary & environmental sciences study design

All studies must disclose on these points even when the disclosure is negative.

Study description	We measured changes to the wing-lengths of moth specimens (>8000 individuals) based on resurvey data for seven assemblages on Mt. Kinabalu, Borneo in 1965 and 2007. We compared the relative contribution of range shift and species size changes to alter community size structure. We found significant size reductions in tropical insects (a mean shrinkage of 1.3% per species over 42 years), but range shifts caused most size re-structuring of assemblages, due to uphill shifts of relatively small species, especially at high elevations. Overall, the mean forewing length of moth assemblages shrank by c.a. 4.9%, of which species range boundary shifts accounted for 3.9%. The remaining variation was due to (within-boundary) distribution changes (0.5%), and within-species size shrinkage (0.6%).
Research sample	We were able to collect forewing length data from 5536 specimens of 277 species in the historical 1965 survey, and 3053 individual specimens from 219 species in the 2007 survey, representing 74.4% to 99.1% of individuals at a site. Female and male individuals were of similar abundance (female = 4122 individuals, male = 4467) and were analyzed separately to account for sexual size dimorphism. Both males and females displayed similar body size change patterns in our preliminary analyses, and so we present results for females only, given that functionally important population-level reproductive output and dispersal are more strongly driven by female than by male morphology (number of female individuals = 2928 and 1224 in years 1965 and 2007, respectively).
Sampling strategy	Chen et al. (2009) resurveyed the moth transect on Mt. Kinabalu in 2007, 42 years after the original study in 1965, using the same field protocols and visiting the same sites along the elevation gradient. We used all specimens available (7 of the 10 sites) from both surveys to compare the size structure changes.
Data collection	Moth specimens were retained from the two field surveys. The 2007 specimens were preserved in National Cheng-Kung University, Taiwan and the 1965 specimens were preserved in the Natural History Museum, London. We measured right forewing length from the wing-thorax junction to wing tip as an index of individual moth body size. All measurements were performed by the same person (CHW) using a dissecting microscope with a 0.01 mm precision digital caliper.
Timing and spatial scale	The transect resurvey includes 10 sites between 1,265 and 3,675 m in Mt. Kinabalu (4095m) and was conducted at the same location and season, August to September 2007, with actual dates at each site following similar moon phases of that in 1965.
Data exclusions	Specimens with damaged or curled wings or where the sex of the specimen had not been identified were excluded.<br>We considered only species presence/absence at sites (assemblages), and not their population abundance at each site, so each species received identical weighting when computing the body size structure of assemblages. Using presence/absence data reduces potential biases associated with stochastic population dynamics during the two survey periods, and is a conservative approach to the analyses. However, to ensure the robustness of our conclusions, we repeated the analyses by weighting species according to their relative abundances at each site in each year, but this did not qualitatively change our conclusions.
Reproducibility	All conclusions were based on random sub-sampling of the data and were reproducible.
Randomization	To detect a shift in assemblage size structure at a site over 42 years, we examined the site-specific change in average body size (the arithmetic mean of site-specific forewing lengths of species in 2007, minus the same metric for 1965) and in coefficient of variance (CV) of site-specific species forewing length (site-specific CV in 2007 minus CV in 1965). To reduce potential sampling bias due to unequal catch sizes in the two surveys, we applied a re-sampling method to provide robust estimate of average body size and CV at each site in both years. At each site, we randomly sub-sampled the individuals to be 80% of the smaller sample size of the two years (modified from Chen et al. (2009). We pooled the results from 500 re-samplings to generate the mean and 95% confidence interval for: 1) the average body size and CV of each site in each year, and 2) changes in average body size and CV between 1965 and 2007 at each site.<br>In order to identify how range shift and intraspecific size change contribute to the new assemblage sizes, we firstly defined four categories of species composition changes that are associated with range shifts of each species between 1965 and 2007, which were expansion or contraction at upper- or lower- range boundaries. We also recognized local extinction, colonization or persistence at sites within each species' elevational limits, and included them in the category of 'non-boundary dynamics'. To partition the variation explained by different processes, we re-computed the 2007 assemblage size by allowing just one of the six processes to occur at a time (intraspecific size change, four categories of range shift, and non-boundary dynamics). We obtained mean and error of each process's contribution to community size structure change from 500 re-samplings of the data set (to account for differences in sample effort between surveys) and interpreted their relative contribution.
Blinding	Not applicable for samples from the field.

Did the study involve field work? ☐ Yes ☒ No

Reporting for specific materials, systems and methods

We require information from authors about some types of materials, experimental systems and methods used in many studies. Here, indicate whether each material, system or method listed is relevant to your study. If you are not sure if a list item applies to your research, read the appropriate section before selecting a response.

Materials & experimental systems

n/a	Involved in the study
☒	☐ Antibodies
☒	☐ Eukaryotic cell lines
☒	☐ Palaeontology
☐	☒ Animals and other organisms
☒	☐ Human research participants
☒	☐ Clinical data

Methods

n/a	Involved in the study
☒	☐ ChIP-seq
☒	☐ Flow cytometry
☒	☐ MRI-based neuroimaging

Animals and other organisms

Policy information about studies involving animals; ARRIVE guidelines recommended for reporting animal research

Laboratory animals	n/a
Wild animals	n/a
Field-collected samples	We measured moth specimens that were retained from the two field surveys (see above). The 2007 specimens were preserved in National Cheng-Kung University, Taiwan and the 1965 specimens were preserved in the Natural History Museum, London. The specimen measurement is approved by the original collectors — I-Ching Chen and Jeremy Holloway, respectively.
Ethics oversight	n/a

Note that full information on the approval of the study protocol must also be provided in the manuscript.
