# Supplementary material for: Reduced body sizes in climate-impacted Borneo moth assemblages are primarily explained by range shifts
Source: Nat Commun. 2019 Oct 10;10:4612. doi: 10.1038/s41467-019-12655-y (PMC6787050; doi:10.1038/s41467-019-12655-y)
Supplement: Supplementary file 2 — Supplementary Information [file 41467_2019_12655_MOESM2_ESM.pdf]

SUPPLEMENTARY INFORMATION

Title: Reduced body sizes in climate-impacted Borneo moth assemblages are primarily explained by range shifts.

Authors: Wu et al.

Supplementary Tables

Supplementary Table 1. Absolute (mm) and percentage change (in parentheses) in average body sizes (forewing length) at each site (from year 1965 to 2007), caused by intraspecific body size change, range boundary shift and non-boundary dynamics. Mean ± 95% confidence interval of both absolute and percentage change are shown. n.a.: not available due to absence of corresponding event at the site. The 'Average' column is the average of the means at each site. Results significant at 95% confidence level are in bold.

	HQ 1440m	PS 1885m	K 2260m	RS 2685m	PC 3085m	PL 3315m	SS 3675m	Average
Species body size change	**-0.11±0.09 (-0.5±0.5%)**	**-0.11±0.02 (-0.6±0.1%)**	**-0.19±0.07 (-1.1±0.4%)**	-0.01±0.04 (-0.1±0.2%)	**-0.23±0.06 (-1.3±0.3%)**	**-0.29±0.12 (-1.7±0.7%)**	**0.23±0.07 (1.3±0.4%)**	**-0.10±0.03 (-0.6±0.2%)**
Range boundary shifts	0.21±1.06 (1.1±5.5%)	**0.34±0.27 (1.8±1.5%)**	-0.06±0.43 (-0.4±2.5%)	**-0.96±0.51 (-5.1±2.7%)**	**-1.49±0.92 (-8.5±4.8%)**	-0.56±0.97 (-3.3±5.7%)	**-2.34±1.06 (-12.9±5.9%)**	**-0.69±0.31 (-3.9±1.7%)**
Non-boundary dynamics	-0.27±0.78 (-1.4±4.0%)	0.28±0.36 (1.5±1.9%)	0.08±0.34 (0.5±2.0%)	**-0.70±0.46 (-3.7±2.4%)**	0.15±0.94 (0.9±5.3%)	0.01±0.61 (0.1±3.7%)	n.a.	-0.09±0.31 (-0.5±1.7%)
Overall	-0.19±1.18 (-0.9±6.1%)	**0.53±0.42 (2.9±2.3%)**	-0.16±0.45 (-0.9±2.6%)	**-1.67±0.67 (-8.9±3.4%)**	**-1.76±1.05 (-10.1±5.4%)**	-0.79±1.10 (-4.6±6.5%)	**-2.13±1.10 (-11.7±6.1%)**	**-0.88±0.35 (-4.9±1.9%)**

Supplementary Table 2. Absolute and percentage change in species forewing length variation (in C.V.) at each altitude site (from year 1965 to 2007) caused by intraspecific body size change, range boundary shift and non-boundary dynamics. Mean ± 95% confidence interval of both absolute and percentage change are shown. Absolute changes in C.V. are shown in $1/10^3$ for clearance. n.a.: not available due to absence of corresponding event at the site. The 'Average' column is the average of the mean estimates at each site. Results significant at 95% confidence level are in bold.

	HQ 1440m	PS 1885m	K 2260m	RS 2685m	PC 3085m	PL 3315m	SS 3675m	Average
Species body size change	1.38±4.29 (0.4±1.4%)	**6.42±1.42 (1.9±0.4%)**	**4.38±3.06 (1.4±1.0%)**	-1.43±1.82 (-0.4±0.5%)	**-7.17±1.59 (-2.6±0.4%)**	**-12.1±5.21 (-3.7±1.6%)**	**-39.5±9.25 (-15.3±0.5%)**	**-6.9±1.7 (-2.2±0.5%)**
Range boundary shifts	49.1±53.9 (16.0±19%)	8.37±14.7 (2.6±4.6%)	**34.2±20.8 (11.0±7.0%)**	**-24.8±19.3 (-7.2±5.7%)**	**56.0±39.9 (20.9±18.2%)**	9.03±31.1 (2.8±9.6%)	**98.8±57.5 (39.8±27.8%)**	**33.0±14.47 (10.7±5.0%)**
Non-boundary dynamics	18.8±29.8 (6.0±9.9%)	**16.7±15.2 (5.1±4.8%)**	1.29±15.6 (0.4±5.0%)	-17.7±18.9 (-5.1±5.4%)	28.4±34.5 (10.5±13.9%)	-3.98±23.6 (-1.2±7.1%)	n.a.	8.55±11.34 (2.8±3.7%)
Overall	**70.5±55.7 (22.8±20.8%)**	**35.8±20.3 (10.9±6.7%)**	**39.7±20.4 (12.8±7.0%)**	**-59.1±26.3 (-17.1±7.1%)**	**77.4±41.0 (28.7±20.1%)**	-4.90±31.3 (-1.46±9.7%)	**72.7±60.1 (29.5±27.1%)**	**33.2±14.62 (10.8±5.1%)**

Supplementary Table 3. Intraspecific altitudinal body size clines. List of species with rank correlation analysis of specimen forewing length against elevation. Species are grouped by the sign of their Kendall's tau correlation metric. Brackets after each species' name show Kendall's tau and sample size (number of specimens). This table only includes the species that were present at more than two sites in 1965 and 2007.

	Species collected in 1965	Species collected in 2007
Positive size cline	*Eupithecia mundiscripta (1, 6)*	*Organopoda cnecosticta (0.86, 12)*
	Collix intrepida (0.82, 14)	*Platycerota percrinata (0.79, 22)*
	Bornealcis versicolor (0.6, 78) [1]	*Bornealcis versicolor (0.52, 74)*
	Phthonoloba stigmalephora (0.6, 12)	*Eupithecia mundiscripta (0.49, 26)*
	Ornithospila succincta (0.55, 8)	*Syncosmia eurymesa (0.48, 34)*
	Cleora mjobergi (0.54, 72)	*Ectropis pais (0.46, 42)*
	Catoria proicyrta (0.53, 28)	*Perixera absconditaria (0.45, 10)*
	Ectropis pais (0.51, 78)	*Bornealcis expleta (0.41, 78)*
	Abraxas invasata (0.48, 34)	*Tympanota erecta (0.38, 94)*
	Pasiphila chlorocampsis (0.43, 52)	*Pasiphila palpata (0.37, 94)*
	Syncosmia eurymesa (0.42, 230)	*Gasterocome pannosaria (0.36, 20)*
	Luxiaria mitorrhaphes (0.42, 34)	*Tympanota arfakensis (0.33, 112)* [3]
	Episteira vacuefacta (0.41, 22)	*Dasyboarmia isorropha (0.26, 12)*
	Cyclophora hirtipalpis (0.33, 6)	*Alcis praevariegata (0.26, 148)*
	Phthonoloba bracteola (0.33, 6)	*Pasiphila chlorocampsis (0.25, 62)*
	Brabira emerita (0.32, 40)	*Tasta montana (0.18, 20)*
	Phthonoloba lutosa (0.31, 32)	*Pogonopygia pavida (0.18, 84)*
	Pasiphila rufogrisea (0.29, 20)	*Eupithecia kamburonga (0.15, 30)*
	Cyclophora carsoni (0.29, 30)	*Tympanota ceramica (0.09, 12)*
	Ecliptoptera furvoides (0.26, 20)	*Poecilasthena nubivaga (0.09, 204)*
	Syncosmia discisuffusa (0.24, 40)	*Hypocometa titanis (0.05, 34)*
	Bornealcis expleta (0.24, 96) [1]	*Syncosmia layanga (0.01, 36)*
	Hypocometa clauda (0.23, 14)	*Myrioblephara simplaria (0, 112)*
	Pasiphila rubrifusa (0.23, 16)	
	Platycerota percrinata (0.22, 36)	
	Hypocometa leptomita (0.2, 60)	
	Tympanota erecta (0.19, 192)	
	Acolutha albipunctata (0.18, 8)	
	Hypocometa titanis (0.18, 188) [2]	
	Orothalassodes glabrosa (0.18, 116)	
	Dasyboarmia isorropha (0.17, 40)	
	Chloroclystis obturgescens (0.16, 94)	
	Cyclophora flavissima (0.16, 36)	
	Synegia obscura (0.15, 60)	
	Synegia ocellata (0.13, 54)	
	Pomasia salutaris (0.12, 146)	
	Orthocabera similaria (0.12, 84)	
	Myrioblephara bifida (0.12, 60)	
	Sauris usta (0.09, 12)	
	Alcis praevariegata (0.07, 312)	
	Platycerota balia (0.06, 14)	
	Ozola submontana (0.04, 152)	
	Perixera absconditaria (0.02, 22)	
	Dindica alaopis (0.02, 138)	
	Luxiaria tephrosaria (0.02, 194)	
	Gasterocome pannosaria (0.01, 64)	
	Pasiphila palpata (0.01, 316)	

Supplementary Table 3. (continued)

	Species collected in 1965	Species collected in 2007
Negative size cline	*Tympanota ceramica (-0.46, 36)*	*Petelia delostigma (-0.55, 8)*
	Axinoptera penataran (-0.41, 16)	*Pomasia sacculobata (-0.06, 56)*
	Sarcinodes reductatus (-0.34, 18)	*Pomasia salutaris (-0.1, 54)*
	Ruttellerona lithina (-0.27, 32)	*Synegia ocellata (-0.02, 22)*
	Eupithecia kamburonga (-0.27, 18)	
	Poecilasthena nubivaga (-0.23, 152) [4]	
	Phthonoloba altissima (-0.18, 8)	
	Myrioblephara simplaria (-0.14, 148)	
	Pelagodes falsaria (-0.11, 138)	
	Conolophia nigripuncta (-0.1, 22)	
	Organopoda cnecosticta (-0.07, 14)	
	Poecilasthena character (-0.05, 74)	
	Cyclophora lowi (-0.05, 46)	
	Synegia decolorata (-0.04, 24)	
	Pasiphila sayata (0, 60) [5]	
	Luxiaria hyalodela (0, 40)	

1. These species belong to this Bornean endemic genus of three. On Kinabalu, *versicolor* replaces *repleta* at higher altitudes and is considerably larger.
2. This species is the largest larentiine in Borneo and is only known from Kinabalu, falling into the highest (summit) association. Its sister species occurs at lower altitudes more widely in Borneo and is distinctly smaller.
3. This species was also abundant in 1965, but the specimens could not be located when the forewing measuring was undertaken.
4. This species, with *titanis* and *sayata*, is characteristic of the summit zone on Kinabalu and is only known from there. It too has a smaller congener at lower altitudes that is not endemic to Borneo.
5. This is the largest species in a genus with 9 species in Borneo, 5 endemic, 4 only known from Kinabalu, and *sayata* is the highest of these.

Supplementary Table 4. Estimating missing body size data. Site-species body size data availability under three estimation approaches and no estimation (Raw) are given in percentages. Percentages with female body size available are shown. Between-year + nearest-site estimation was used to produce the results in the main text.

Site<br>Elevation		HQ<br>1440m	PS<br>1885m	K<br>2260m	RS<br>2685m	PC<br>3085m	PL<br>3315m	SS<br>3675m
Number of species	1965	212	220	103	65	9	7	5
recorded	2007	90	170	103	68	8	10	5
Raw (no estimation)	1965	62.3%	72.7%	68.9%	72.3%	66.7%	85.7%	80.0%
	2007	39.6%	50.0%	60.2%	49.3%	87.5%	90.0%	60.0%
Between-year	1965	63.2%	75.9%	73.8%	80.0%	77.8%	85.7%	80.0%
estimation only	2007	60.4%	66.3%	72.8%	68.1%	87.5%	90.0%	60.0%
Nearest-site estimation	1965	75.5%	80.9%	90.3%	96.9%	88.9%	100%	100%
only	2007	62.6%	56.4%	84.5%	79.7%	100%	100%	100%
Between-year +	1965	77.4%	82.7%	92.2%	98.5%	100%	100%	100%
nearest-site estimation	2007	73.6%	69.8%	90.3%	92.8%	100%	100%	100%

SUPPLEMENTARY FIGURES

Supplementary Figure 1. Species body size change in geometrid moths on Mt. Kinabalu over a 42-year study period (1965 vs. 2007). This analysis calculated the arithmetic mean of forewing length measured from individual specimens (number of individuals included = 3479, from 109 species with size data in both surveys). Linear mixed model shows body sizes of individual species decreased by 1.3% on average (mean ± SE shrinkage = 0.25 ± 0.04 mm; $t = -6.37$, $P < 0.001$).

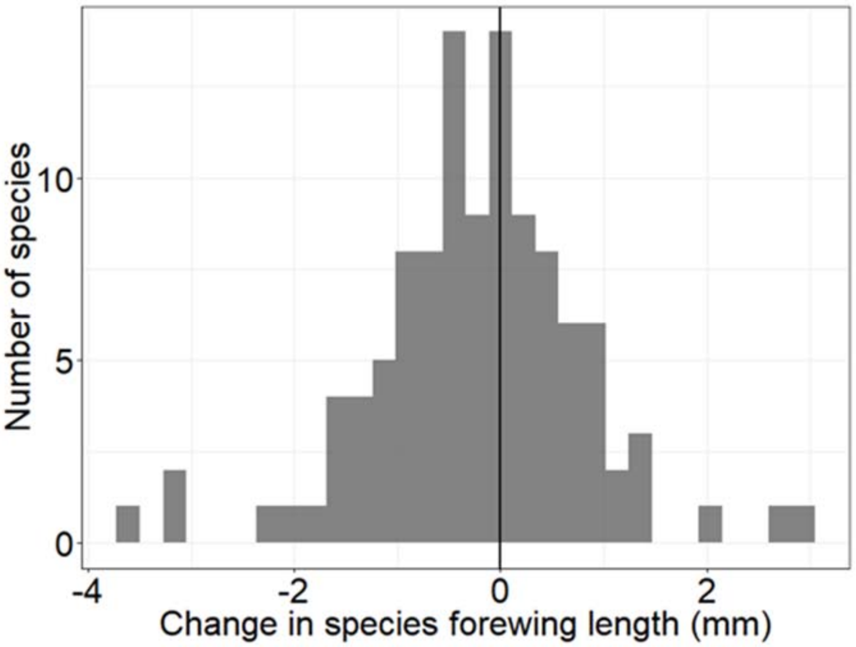

Supplementary Figure 2. Contributions of intraspecific body size change and species composition change (induced by four types of range boundary shift and non-boundary dynamics) to (a) average body size and (b) species body size variation (coefficient of variation) of geometrid moth assemblages across elevation over 42 years, based on the average of 500 runs of re-sampling. Asterisks indicate components with effect size significantly different from zero at the 95% confidence level.

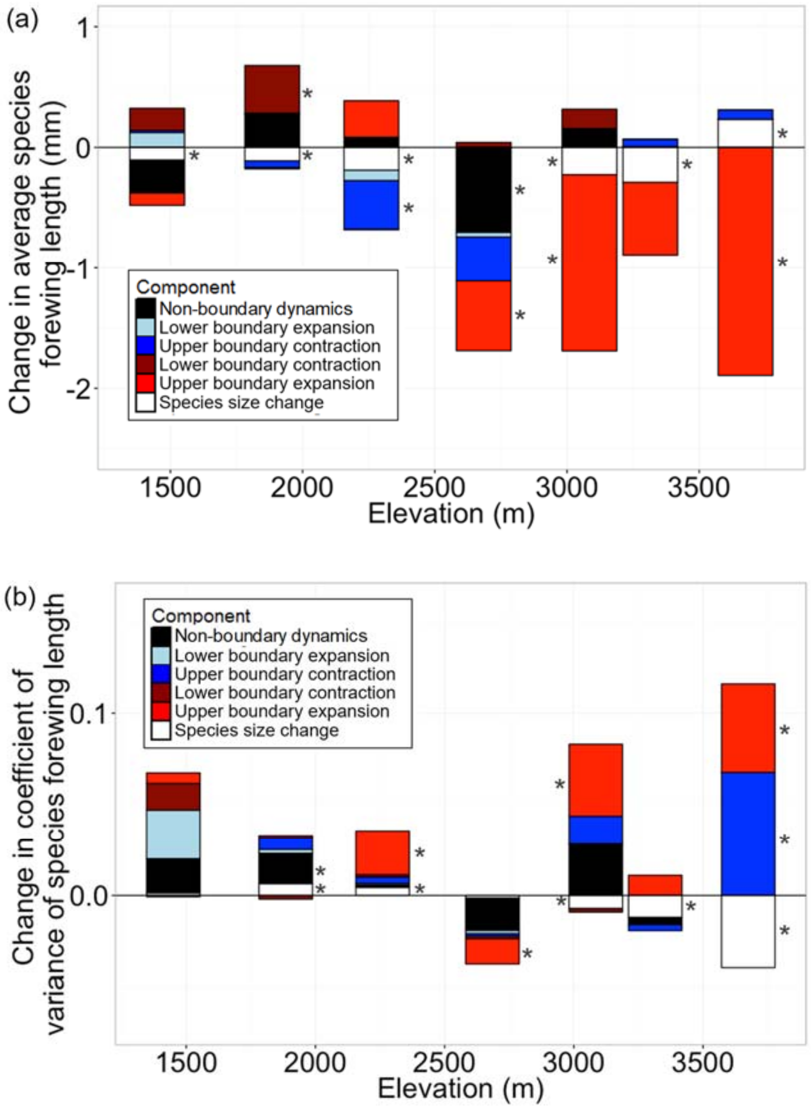

Supplementary Figure 3. Moth assemblage size structure in 1965 (black) and 2007 (red) based on weighting species by their abundances at each site. (a) Average forewing length (mm). (b) Coefficient of variation of species forewing length. (c) Frequency distribution of species forewing length. In (a) and (b), mean and 95% confidence intervals at each site are shown, based on 500 resamples. Data points are overlaid. Asterisks indicate significant ($p<0.05$) differences between 1965 and 2007. In (c), number of species are on log10 scale and overlaps between the two years are illustrated in grey.

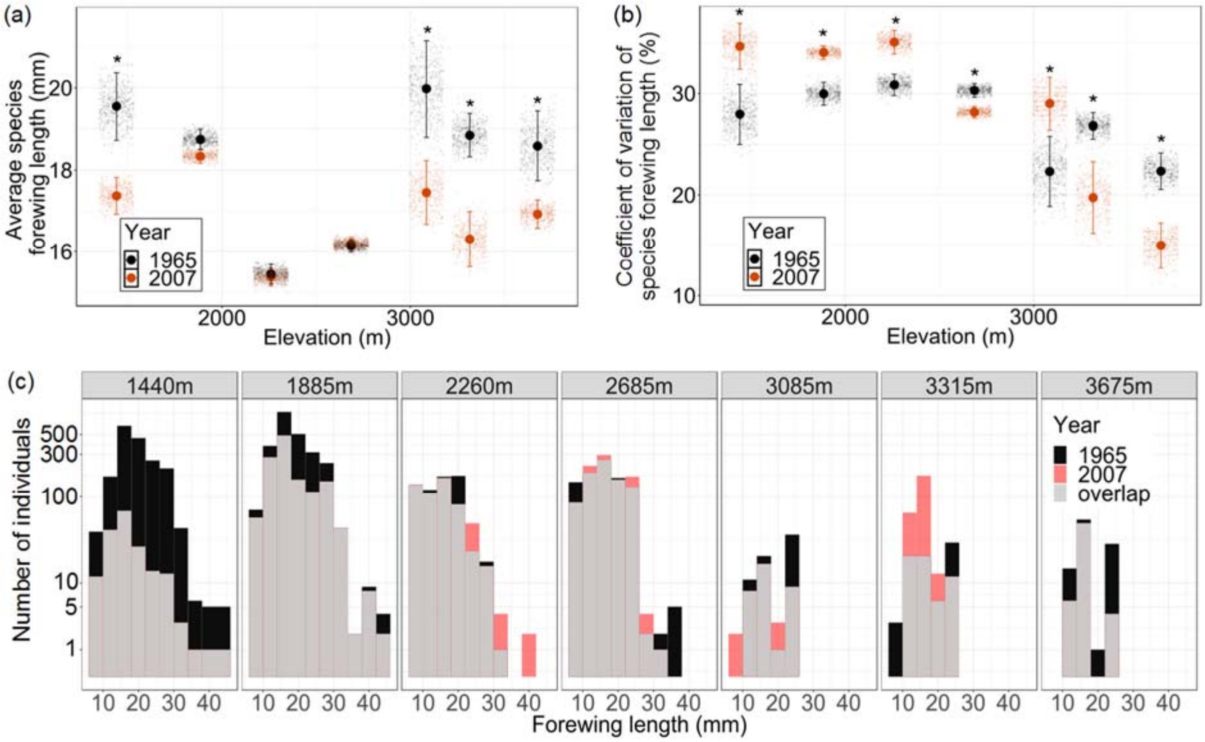

Supplementary Figure 4. Contribution of range boundary shifts (black), intraspecific size change (white) and non-boundary dynamics (grey) to changes in moth assemblage size structure from 1965 to 2007, based on weighting species by their abundances at each site. (a) Change in average species forewing length. (b) Change in coefficient of variation of species forewing length. Asterisks indicate components with effect sizes significantly different from zero at the 95% confidence level, based on 500 resamples.

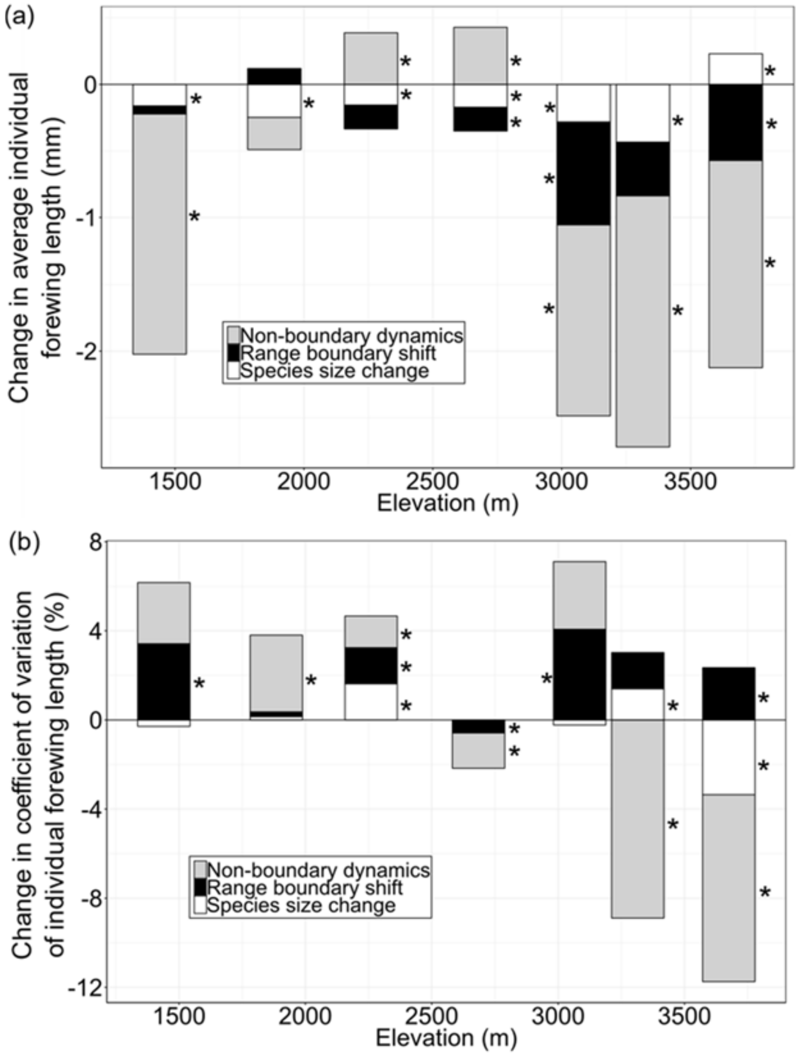

Supplementary Figure 5. Species body size change vs. species body size in 1965. Each dot represents a single species. Body size is the arithmetic mean forewing length of all female specimens in each year. Kendall's rank correlation is used with sample size (N), tau-b, and p-value reported.

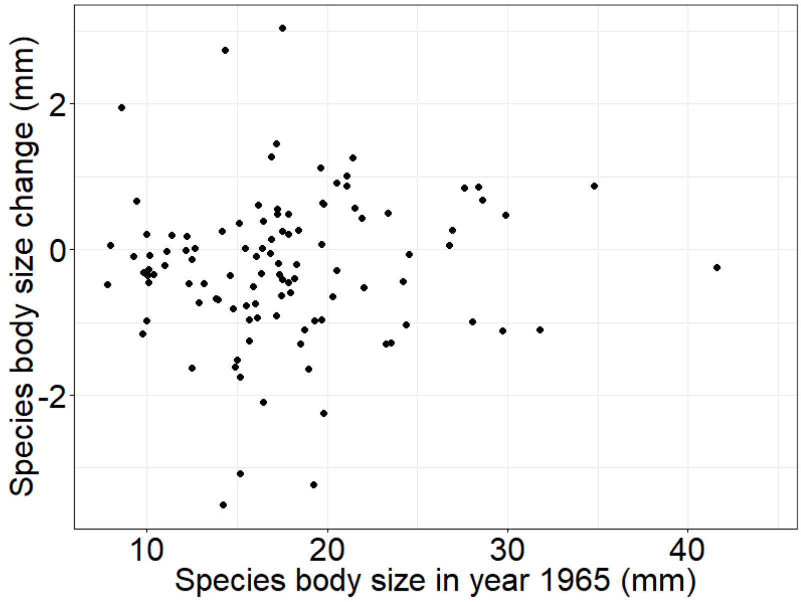

	N	Kendall's tau-b	p-value
Species body size change vs. species body size in 1965	106	0.082	0.21

Supplementary Figure 6. Intraspecific body size clines with elevation for individual species, based on Kendall's rank correlations of specimen forewing length against elevation. We only include species that were present at more than two sites in 1965 or 2007 (N = 63 and 27 in 1965 and 2007, respectively). Positive Kendall's tau indicates positive body size cline with elevation.

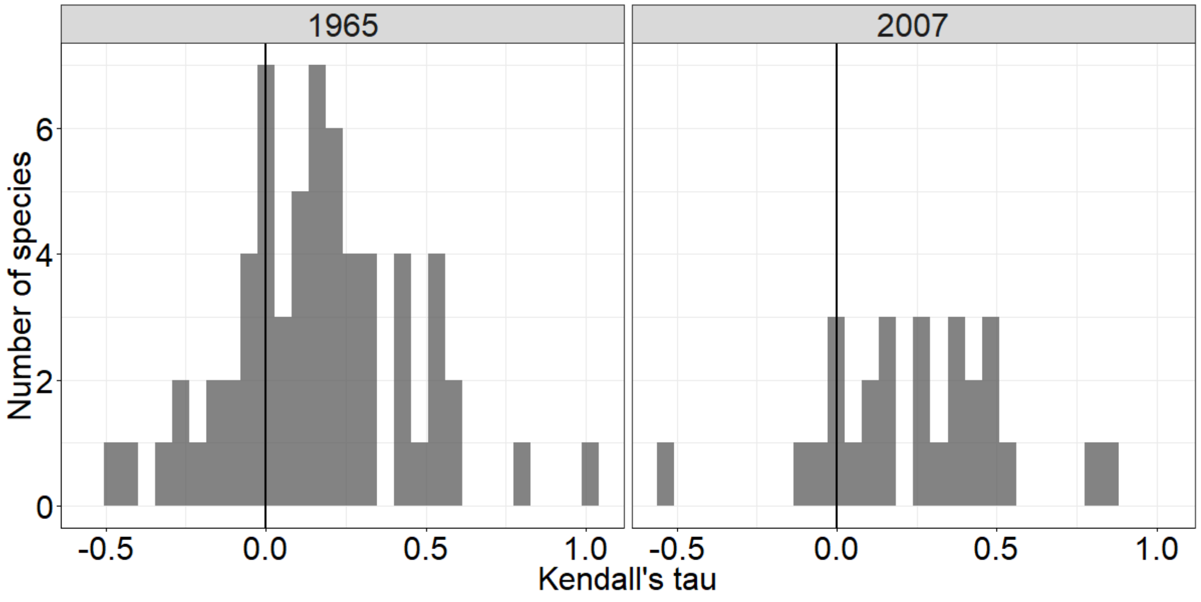

Supplementary Figure 7. Interspecific body size clines. Relationships are shown between the body size (forewing length) of each species versus its upper range boundary. Dashed and solid lines indicate insignificant and significant slopes in ordinary linear regressions (using *lm*() function in R), respectively. The rare subfamilies Oenochrominae and Desmobathrinae were excluded.

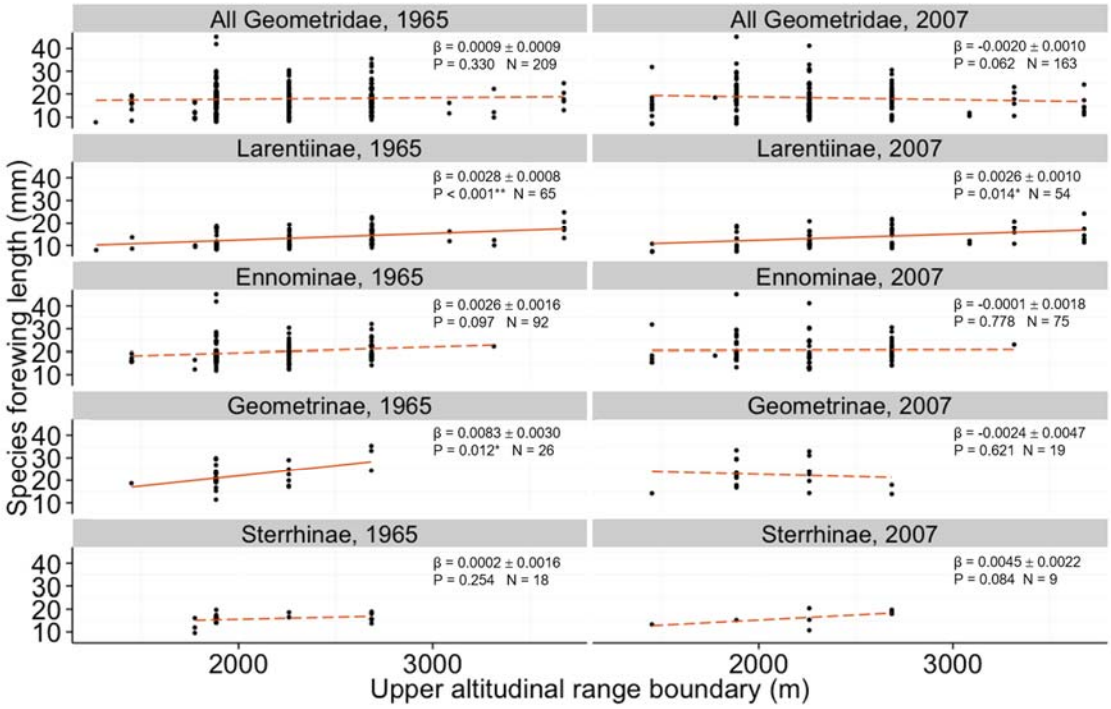

Supplementary Figure 8. Shifts in (a) upper and (b) lower range boundary elevation vs. species body size in 1965. Each dot represents a single species. Body size is the arithmetic mean forewing length of all female specimens in each year. Kendall's rank correlation is used with sample size (N), tau-b, and p-value reported.

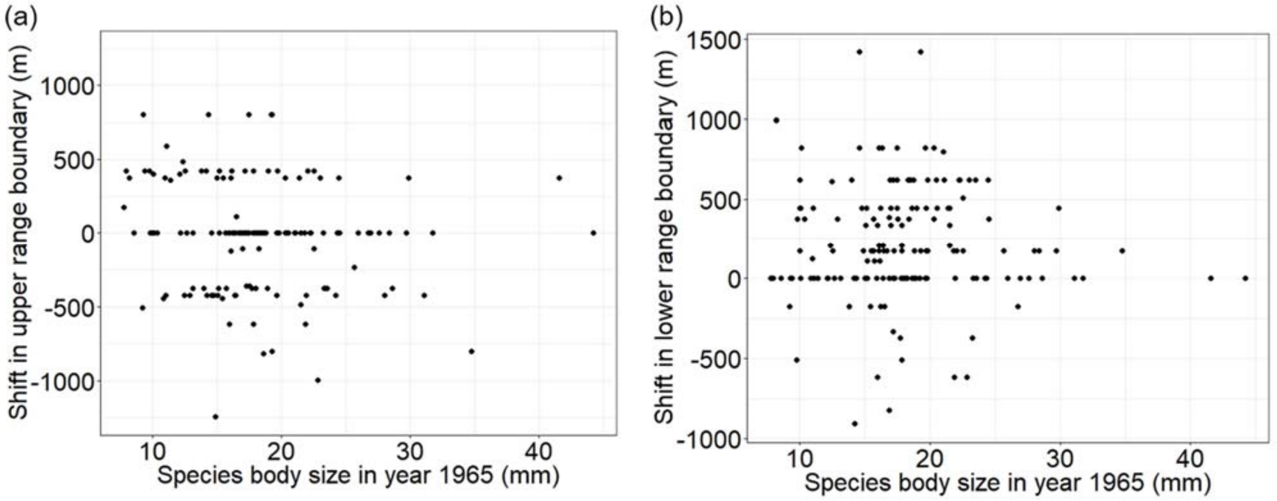

	N	Kendall's tau-b	p-value
Upper boundary shift vs. 1965 body size	156	-0.099	0.09
Lower boundary shift vs. 1965 body size	156	0.031	0.59

Supplementary Figure 9. Shifts in (a) upper and (b) lower range boundary elevation vs. species body size change over the 42 years. Each dot represents a single species. Body size is the arithmetic mean forewing length of all female specimens in each year. Kendall's rank correlation is used with sample size (N), tau-b, and p-value reported.

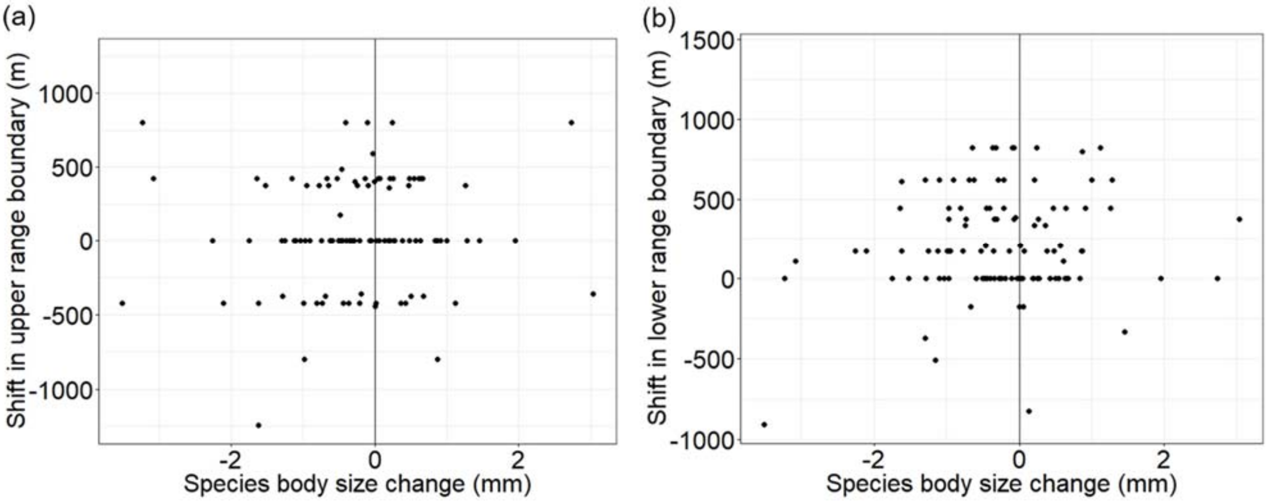

	N	Kendall's tau-b	p-value
Upper boundary shift vs. body size change	108	0.065	0.363
Lower boundary shift vs. body size change	108	0.031	0.657

Supplementary Figure 10. Contributions of intraspecific body size change in narrowly- and widely distributed species (categorized according to number of sites occupied in 1965) to assemblage body size changes in terms of (a) average body size and (b) coefficient of variance (CV) of body size of geometrid moths along the elevation gradient over the 42-year study period. Data points are overlaid. Asterisks indicate components with effect sizes significantly different from zero at the 95% confidence level, based on the 500 runs of re-sampling.

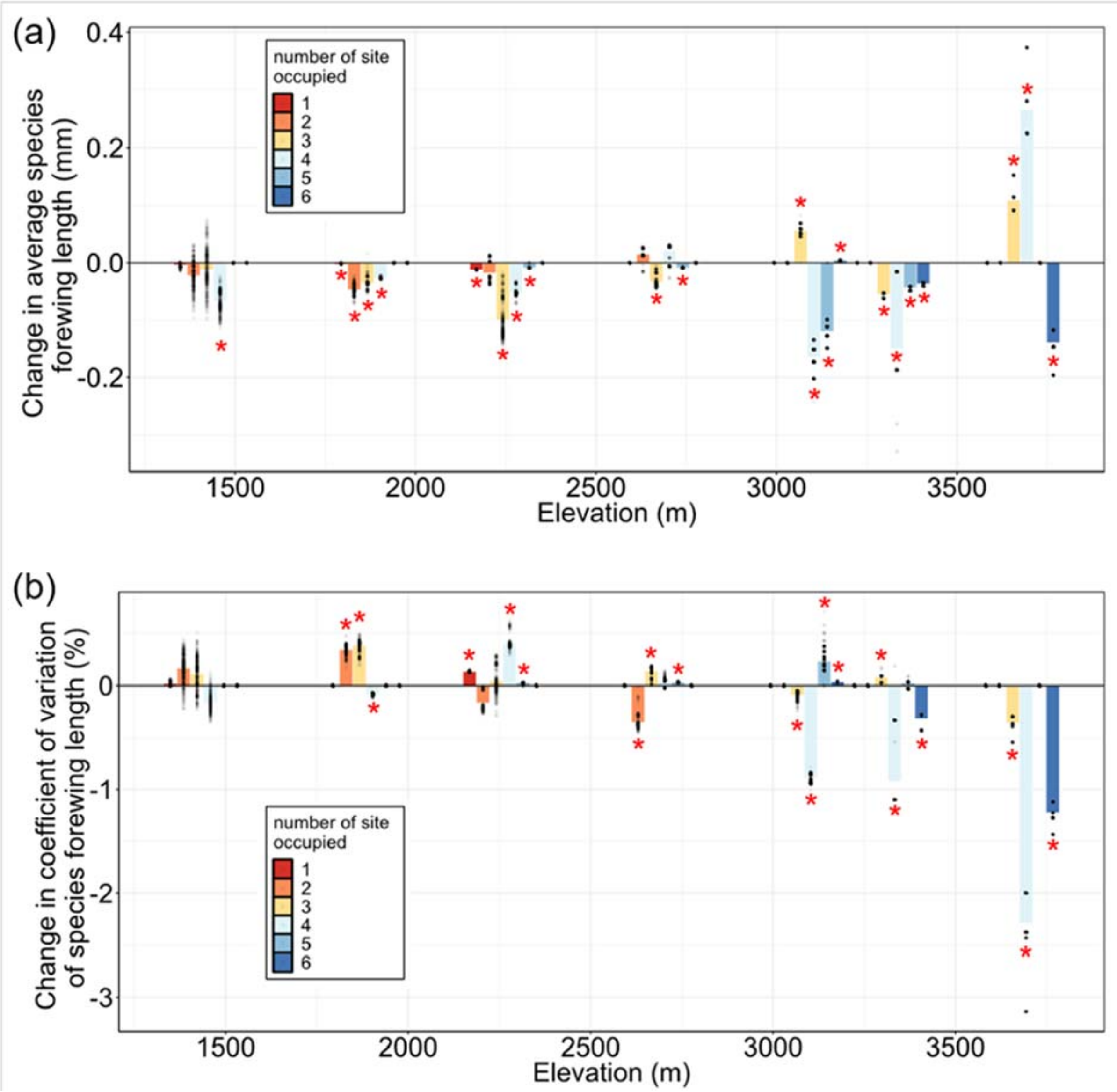

Supplementary Figure 11. Elevational distribution range of Mt. Kinabalu geometrid species present in 1965, and their distribution range in 2007. ID: species ranked by their upper range boundary elevation in 1965. Light green shading is to facilitate between year comparison. Distribution data from all 10 sites in the original surveys are used. Dots indicate species that are only detected at one single elevation.

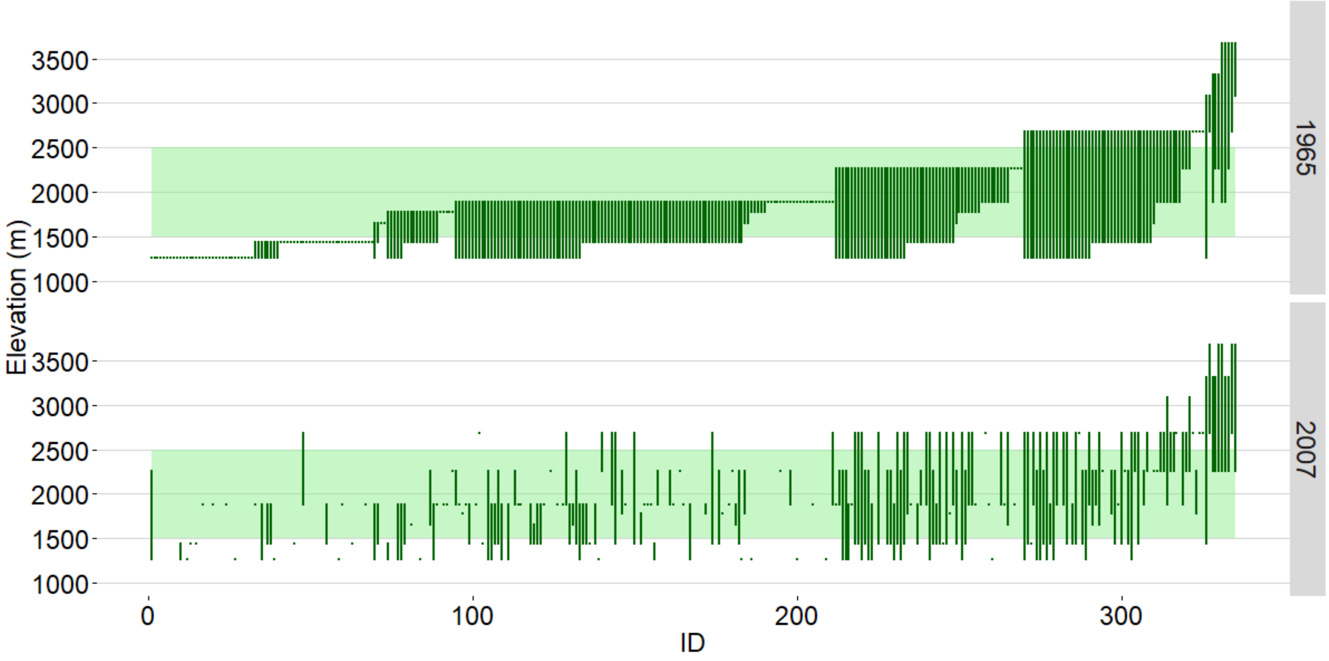

Supplementary Figure 12. Elevation range size vs. species body size in 1965. Each dot represents a single species. Body size is the arithmetic mean forewing length of all female specimens in 1965. Kendall's rank correlation is used with sample size (N), tau-b, and p-value reported.

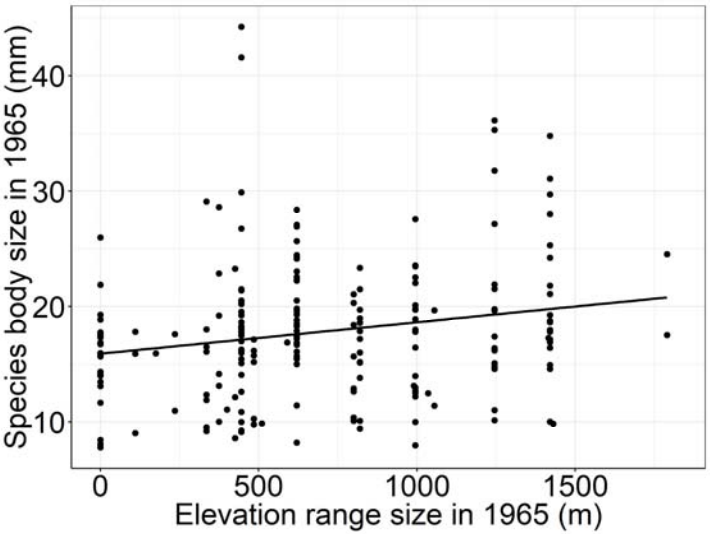

	N	Kendall's tau-b	p-value
Elevation range size vs. species body size in 1965	206	0.151	<0.01

Supplementary Figure 13. Contributions of range boundary shifts in narrowly- and widely-distributed species (categorized according to number of sites occupied in 1965) to assemblage body size changes in terms of (a) average body size and (b) coefficient of variance (CV) of body size of geometrid moths along the elevation gradient over the 42-year study period. Data points are overlaid. Asterisks indicate components with effect sizes significantly different from zero at the 95% confidence level, based on the 500 runs of re-sampling.

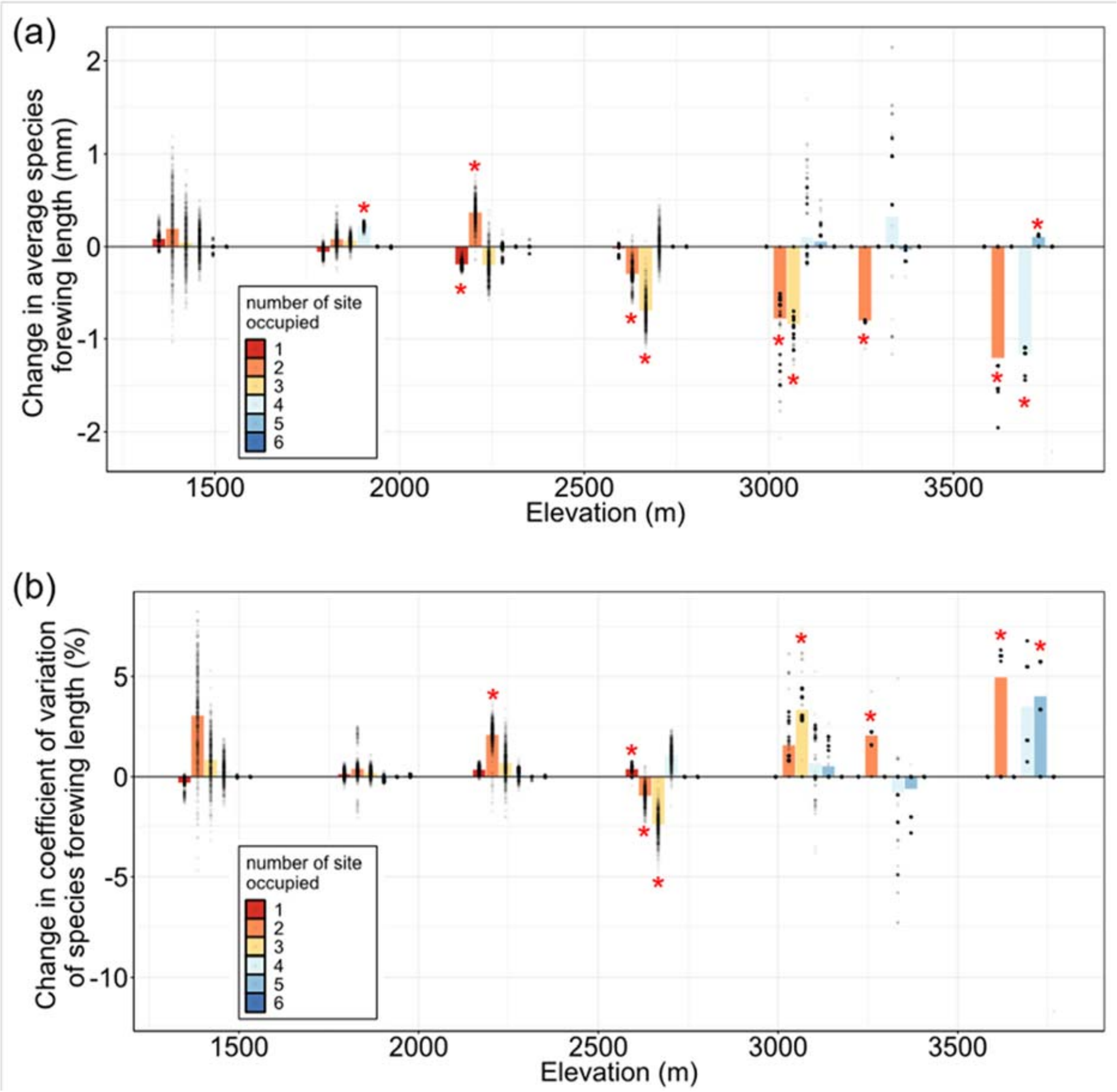

Supplementary Figure 14. Using raw data (i.e. no missing data estimation) to calculate moth assemblage size structure in 1965 (black) and 2007 (red). (a) Average forewing length (mm). (b) Coefficient of variation of species forewing length. (c) Frequency distribution of species forewing length. In (a) and (b), mean and 95% confidence intervals at each site are shown, based on 500 resamples. Data points are overlaid. Asterisks indicate significant ($p<0.05$) differences between 1965 and 2007. In (c), number of species are on log10 scale and overlaps between the two years are illustrated in grey.

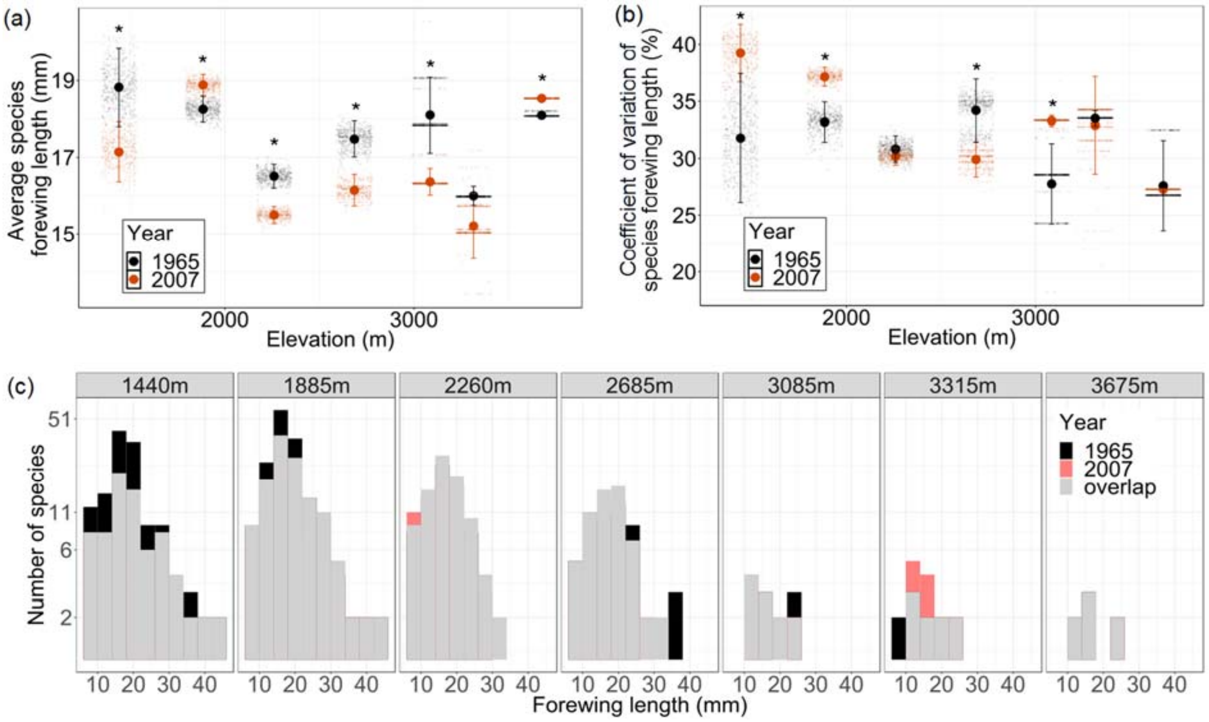

Supplementary Figure 15. Using raw data (i.e. no missing data estimation) to calculate contribution of range boundary shifts (black), intraspecific size change (white) and non-boundary dynamics (grey) to changes in moth assemblage size structure from 1965 to 2007. (a) Change in average species forewing length. (b) Change in coefficient of variation of species forewing length. Asterisks indicate components with effect sizes significantly different from zero at the 95% confidence level, based on 500 resamples.

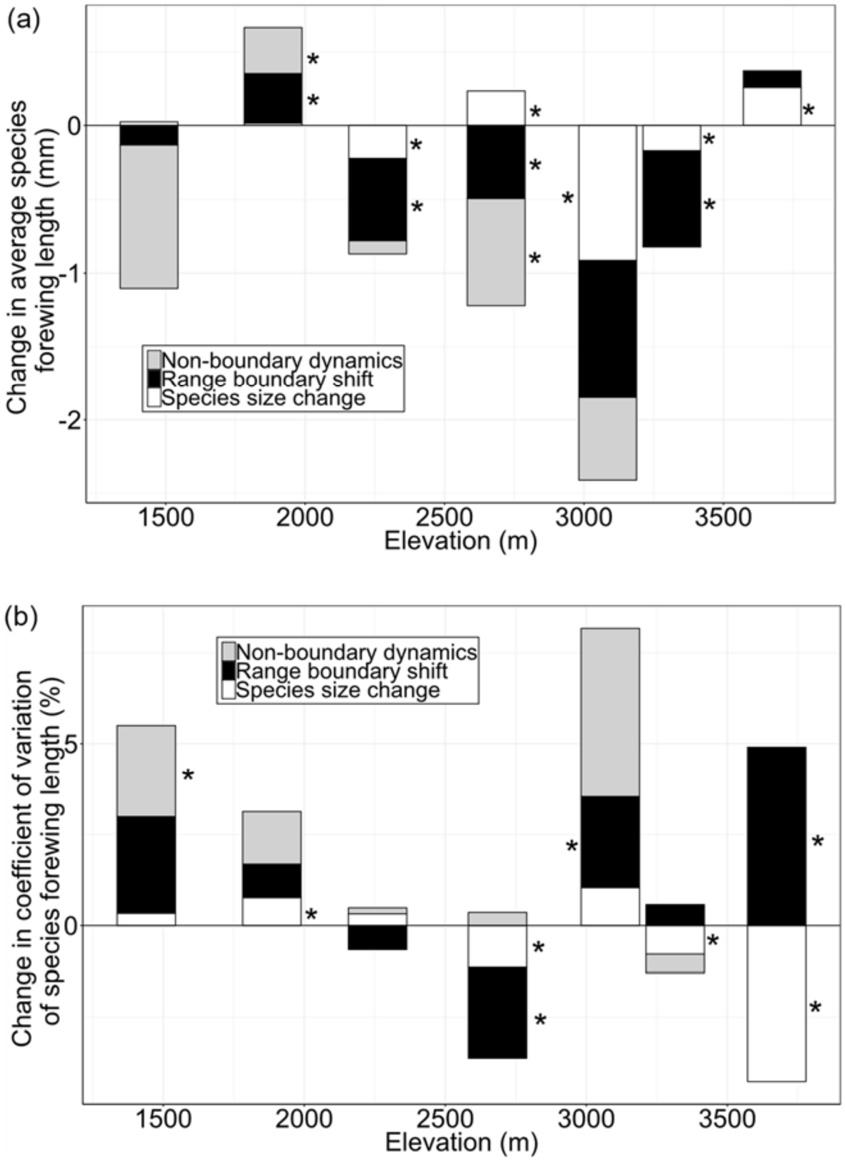

Supplementary Figure 16. Including missing data (nearest-site estimation only) to calculate moth assemblage size structure in 1965 (black) and 2007 (red). (a) Average forewing length (mm). (b) Coefficient of variation of species forewing length. (c) Frequency distribution of species forewing length. In (a) and (b), mean and 95% confidence intervals at each site are shown, based on 500 resamples. Data points are overlaid. Asterisks indicate significant ($p<0.05$) differences between 1965 and 2007. In (c), number of species are on log10 scale and overlaps between the two years are illustrated in grey.

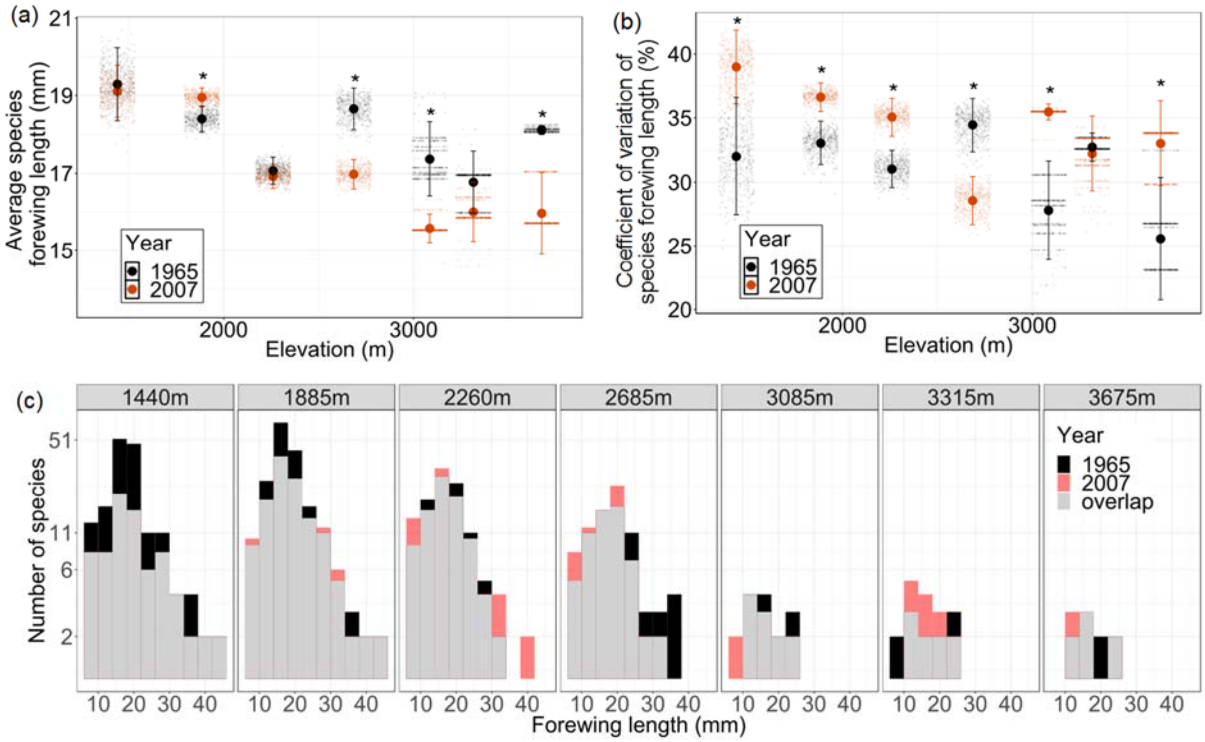

Supplementary Figure 17. Including missing data (nearest-site estimation only) to calculate contribution of range boundary shifts (black), intraspecific size change (white) and non-boundary dynamics (grey) to changes in moth assemblage size structure from 1965 to 2007. (a) Change in average species forewing length. (b) Change in coefficient of variation of species forewing length. Asterisks indicate components with effect sizes significantly different from zero at the 95% confidence level, based on 500 resamples.

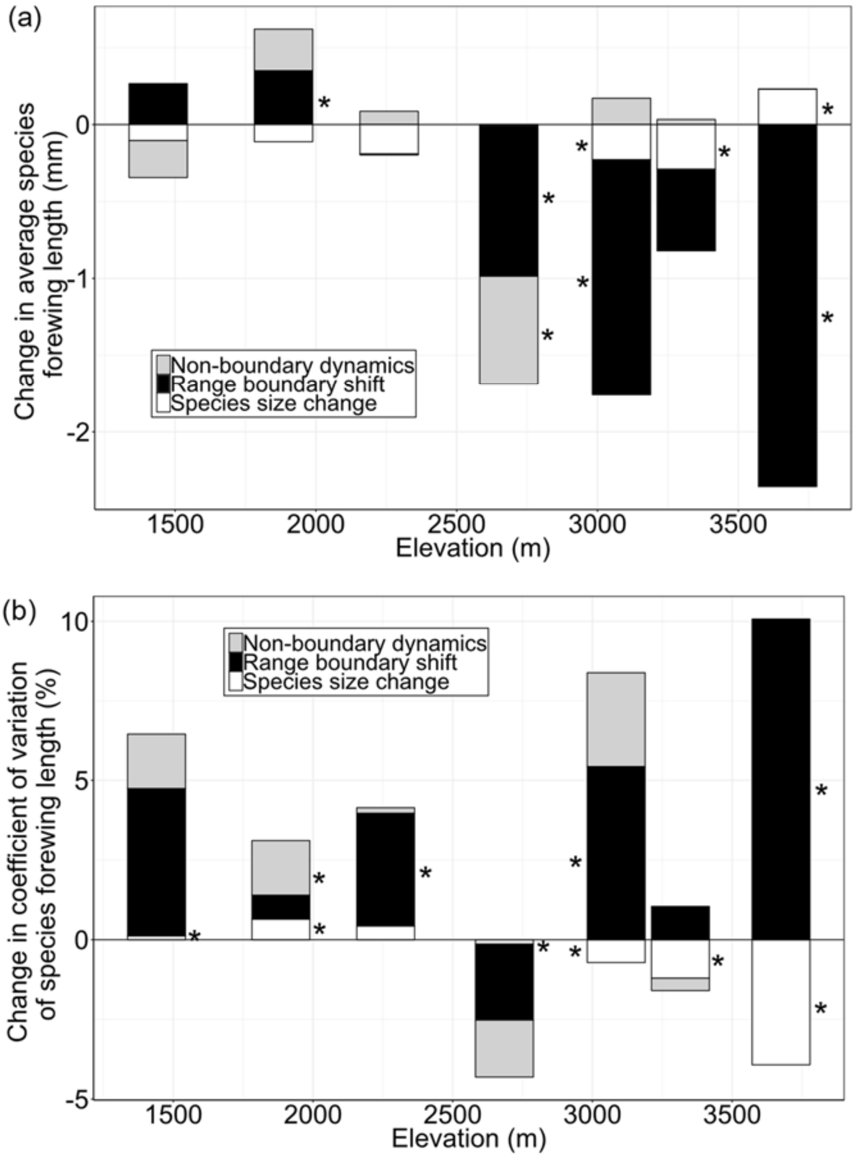

SUPPLEMENTARY NOTE 1

Missing data estimation

<u>Missing body size data estimation</u>

When forewing length of a species at a site is not available (e.g. damaged or missing specimens; 417 out of the 1079 species-site combinations), we conducted a two-step estimation for missing data based on the 662 species-site combinations we had measured. First, the site-specific forewing length of a particular species in one year, if available, was used to estimate the missing data of that species in the other year. Second, if not available, the forewing length of that species from the nearest elevation site(s) in the same year was used to estimate the missing data. An interpolation was applied if two nearest elevation sites were both available (e.g. adjacent higher and lower sites). Our estimation method increased the measured/collected specimens from 662 to 893 out of 1079 species-site combinations, improving the ratio of measured/collected specimens (females) at each site from 39.6 – 90.0% (raw data) to 69.8 – 100% (Supplementary Table 4).

<u>Robustness of results applying missing body size data estimation</u>

To ensure that the results and conclusions drawn in this paper are robust under our missing data estimation approaches, we conducted the same set of analyses on assemblage body size structure (i.e. changes in average and variation in species body size, and contributions by species range shift and species body size change), using 1) raw and 2) nearest-site estimation

only data sets (Supplementary Figure 14-17). We found qualitatively consistent patterns, regardless of the type of data set used, that average body size of assemblages was reduced at higher elevation, there was increasing species body size variation at most sites, and relatively stronger contribution of range shifting, in comparison to size shrinkage, in determining assemblage body size structure. Given that our results were robust to the different ways of considering missing data, we only report results including missing data estimation in the main text.

SUPPLEMENTARY NOTE 2

Correlation between body size, distribution range and their changes

Under climate warming, both species range shift and intraspecific body size change are the results of direct (e.g. metabolic) and/or indirect (e.g. habitat, nutrition) thermal pressure. The two responses by species may interact and compensate for each other if they are responses to similar pressure types (e.g. range shift reduces the metabolic pressure on size shrinkage). Here we examine whether species' shifts at range boundaries correlate with the extent of intraspecific size change. We found no correlation between species size, species size change, and species' range boundary shift over 42 years (Supplementary Figure 5, 8, 9).

Note there are a few caveats in our data set in using it to examine these correlations. First, the lower boundary of our altitudinal transect is chosen arbitrarily. As a result, elevation distributions of moth species recorded at the lowest sites may not represent the species' true pattern in the field. Also, we only examined the forewing lengths of the moth specimens, whereas physiological compensation under range shifting may occur on other traits such as body mass. More detailed surveys and measurements would be needed to investigate these correlations in greater detail, which is beyond the scope of this study.

Altitudinal body size clines may exist not only at the interspecific level (i.e. Bergmann's rule) but also at the intraspecific level, reflecting local adaptation or phenotypic plasticity. If

intraspecific body size clines exist in the Mt. Kinabalu geometrid moth fauna, they could indicate responsiveness in body size of the species to ambient temperature through plasticity or adaptability, and therefore a capability to contribute to size structure change under warming (i.e. via size shrinkage).

Intraspecific body size clines

Here we examined which of the geometrid moth species in this study exhibit intraspecific body size clines (either positive or negative). To ensure statistical robustness, we focused this analysis on a species subset that only includes the species that were present at more than two sites in 1965 and 2007 (number of species = 63 and 27, respectively). The number of sites varied between 3 and 5 (median 3) per species in 1965, and 3 and 4 (median 3) in 2007. For each species, we used Kendall's rank correlation to analyze how forewing length of individual female specimens collected in 1965 or 2007 (raw measurement data) correlate with elevation.

Results show there were more species with positive rank correlations than those with negative ones, in both 1965 and 2007 (Supplementary Figure 6, Supplementary Table 3). We do not report the statistical significance of the rank correlations because small sample sizes in some of the species may bias the results (Supplementary Table 3). A body-size cline with

altitude, if it exists in a species, is indicative of a capacity for size responses to temperature differences. Whether a cline results from physiological adjustment or long-term evolutionary adaptation requires further analysis, and is beyond the scope of this study.

Interspecific body size clines

Interspecific body size clines with altitude, if they exist, would indicate how range boundary shifts may alter community body size structure due to colonisation of smaller or larger species from neighboring locations. We use linear regression to correlate species' upper range boundary elevations with their mean body size in each year, for each moth subfamily and for each subfamily as a whole (excluding the rare Oenochrominae and Desmobathrinae) (Supplementary Figure 7). Positive clines existed in the Larentiinae and Geometrinae subfamilies in 1965 (i.e. consistent with Bergmann's rule), and may indicate range shift impacts on community body size structure (see discussion in main text). Range boundary shifts over the 42 years removed the cline in Geometrinae; this subfamily is not present at the top three sites. No significant clines were found at the family level.

Interested readers may refer to Holloway (1970,1997) [1,3] for taxonomic and biogeographic discussion regarding inter-specific body size pattern with elevation for geometrid moths on Mt Kinabalu. At least nine monophyletic species sister-pairs and one triplet were identified where the size distribution is clinal, with the larger species being the higher. It is also notable

that the geometrid species characteristic of the top three sites, together with two noctuoids, are the largest in their genera in Borneo (see also footnotes to Supplementary Table 3) and all are only found on the mountain. A more analytical approach is being considered for the fauna, particularly the Larentiinae. A more general biogeographic context for montane Lepidoptera in the Indo-Australian tropics was provided by Holloway (1986) [2], noting the unique features of Borneo, though this was before a taxonomic revision [3] when these features were found to be more marked.

SUPPLEMENTARY REFERENCES

1. Holloway, J. D. (1970) The biogeographical analysis of a transect sample of the moth fauna of Mt. Kinabalu, Sabah, using numerical methods. Biol. J. Linn. Soc. 2, 259–286.
2. Holloway, J.D. (1986) Origins of the Lepidoptera faunas of high mountains in the Indo-Australian tropics. In: Vuilleumier, F. & Monasterio, M. (eds.) High altitude Tropical Biogeography, pp. 533-556. Oxford University Press, New York.
3. Holloway, J. D. (1997) The Moths of Borneo: family Geometridae, subfamilies : Sterrhinae, Larentiinae. Malayan Nature Journal 51: 1-242.
